# DDIT4/mTOR signaling pathway mediates cantharidin-induced hepatotoxicity and cellular damage

**DOI:** 10.3389/fphar.2024.1480512

**Published:** 2024-11-05

**Authors:** Wenchao Tang, Yue Pan, Can Zhu, Didong Lou, Fang Peng, Qin Shi, Yuanyuan Xiao

**Affiliations:** ^1^ School of Basic Medicine, Guizhou University of Traditional Chinese Medicine, Guiyang, China; ^2^ School of Traditional Chinese Medicine Health Preservation, Guizhou University of Traditional Chinese Medicine, Guiyang, China

**Keywords:** cantharidin, liver injury, DDIT4, mTOR signaling pathway, autophagy, apoptosis

## Abstract

**Background:**

Cantharidin (CTD) extracted from the traditional Chinese medicine *Mylabris* has significant therapeutic effects on various tumors. However, the high toxicity of CTD can cause serious liver damage, although the related molecular mechanisms remain unclear.

**Methods:**

In this study, we established models of CTD-induced liver and L-O2 cell damage in mice *in vivo* and *in vitro*. Subsequently, liver function indicators were detected in mouse serum, while liver tissues were subjected to pathological and transmission electron microscopy observations. L-O2 cell activity was investigated using the CCK-8 assay, and the mRNA and protein expression of DNA damage-induced transcription factor 4 (DDIT4) in liver tissue and L-O2 cells was detected using qPCR, immunohistochemistry, and western blotting. Western blotting was also used to detect the expression levels of autophagy- and apoptosis-related proteins in liver tissue and L-O2 cells. After RNAi interference with DDIT4, Rap, and 3-MA treatment, autophagy and apoptosis of L-O2 cells were detected using western blotting, flow cytometry, transmission electron microscopy, and confocal microscopy.

**Results:**

Following CTD exposure, the mouse liver showed significant pathological damage and an increase in autophagic lysosomes, while the vitality of L-O2 cells showed a significant decrease. CTD led to a significant increase in the mRNA and protein levels of DDIT4 in both liver tissue and L-O2 cells, as well as a significant increase in LC3-II, Beclin1, and Bax, whereas p-mTOR and Bcl-2 were significantly decreased. Following DDIT4 interference and 3-MA treatment, the levels of autophagy and apoptosis induced by CTD in L-O2 cells were reduced. After Rap treatment, both autophagy and apoptosis of CTD-induced L-O2 cells were significantly enhanced.

**Conclusion:**

The molecular mechanism of CTD-induced toxicity in mouse liver and L-O2 cells is mainly through DDIT4/mTOR signaling pathway activation, leading to an increase in autophagy and apoptosis levels.

## 1 Introduction

*Mylabris*, a traditional Chinese medicine, is the earliest discovered anti-tumor insect drug and has a history of more than 2000 years. *Mylabris* is the dried body of *Mylabris phalerata Pallas* or *Mylabri scichorii Linnaeus* and was first recorded in the “Shen Nong’s Herbal Classic” (Wang, 1989; Wang G. et al., 2018). Cantharidin (CTD) is the main active ingredient extracted from *Mylabris*. Modern pharmacological research has demonstrated that CTD has a strong killing effect on liver, lung, gastric, bladder, breast, and pancreatic cancers, as well as other stubborn tumors (Naz et al., 2020; Ren and Kinghorm,2021). Several Mylabris-/CTD-based drug formulations are available in the Chinese market, including Aidi injection, compound Mylabris capsules, and disodium cantharidinate and vitamin B6 injection injection (Wang J. et al., 2018; He et al., 2021; Huang et al., 2023). However, CTD has strong toxic side effects, long-term clinical application will also lead to adverse reactions, and even death in severe cases (Zhang et al., 2020a; Jin et al., 2023), which greatly limit its further clinical application (Wang G. et al., 2018; Moye et al., 2014).

Numerous studies have shown that CTD can cause toxic damage to multiple organs, including the liver, kidneys, heart, lungs, gastrointestinal tract, bladder, and testes, with liver damage being the most severe (Wang J. et al., 2018; Naz et al., 2020; Chang et al., 2008; Huang et al., 2021; Xiao ea al., 2023). CTD can cause punctate necrosis, vacuole, and edema of rat liver cells, mainly by interfering with lipid metabolism and the steroid hormone biosynthesis pathway (Zhang et al., 2020b). CTD may inhibit the hepatotoxicity induced by drug metabolism by inhibiting the expression of metabolic enzymes in the rat liver (Zhou et al., 2015). Zhu et al. (2019) found that 14 days after intragastric administration of CTD, 54 metabolites and 14 metabolic pathways were disordered, mainly by affecting amino acid and energy metabolism pathways, leading to hepatocyte apoptosis and necrosis. CTD has also been shown to induce cell damage in L-O2 cells with a “dose-time-toxicity” relationship. Dilation of the endoplasmic reticulum, autophagosomes, and apoptotic bodies has been observed under transmission electron microscopy, which further induced apoptosis by regulating oxidative stress, endoplasmic reticulum stress, and autophagy (Liu et al., 2020a). CTD can also induce hepatotoxicity by inhibiting the metabolism of cysteine, methionine, glutathione, glycine, serine, and threonine in L-O2 cells (Liu et al., 2020b). Additionally, we have previously shown that CTD can cause pathological damage and oxidative stress in the liver of mice, mainly by regulating glycerol phospholipid metabolism, the ABC transporter pathway, and choline metabolism (Huang et al., 2021). However, the detailed molecular mechanism of CTD-induced hepatotoxicity remains unclear.

DNA damage-induced transcription factor 4 (DDIT4), also known as DNA damage response-1 (REDD1) or dexamethasone-induced gene-2 (Dig2), is a recognized cellular stress protein that is highly expressed under conditions of hypoxia, DNA damage, and oxidative stress and can negatively regulate the activity of mammalian target of rapamycin (mTOR) protein (Ellisen, 2005; Pan et al., 2023). Many studies have shown that overexpression of DDIT4 can induce autophagy and apoptosis of nerve cells and cardiomyocytes (Li et al., 2017; Huang et al., 2019; Li et al., 2023). DDIT4 plays an important role in autophagy and apoptosis in methamphetamine-induced cardiomyocyte injury and cerebral ischemia-reperfusion injury by regulating the mTOR pathway (Chen et al., 2016; Xu et al., 2021). However, its role in CTD-induced hepatotoxicity has not been reported.

In this study, we established CTD-induced liver injury models in mice and CTD exposure models in normal human L-O2 liver cells through *in vivo* and *in vitro* experiments, respectively, to investigate CTD-induced autophagy and apoptosis in liver cells. Exploring the important role of the DDIT4/mTOR pathway in CTD-induced liver injury and L-O2 cell injury in mice at the pathological, biochemical, molecular, and cellular levels will provide important theoretical references for further elucidating the mechanism of CTD-induced liver toxicity.

## 2 Materials and methods

### 2.1 Chemicals, animals, and treatment

Cantharidin (purity ≥ 98%) was purchased from Sigma Company (USA). According to our previous method (Liu et al., 2023), a CTD suspension (4 mg/mL) was prepared with 0.5% sodium carboxymethyl cellulose solution and stored at 4°C for later use.

Forty SPF grade 6-week-old male Kunming mice, body weight 20 ± 2 g, were purchased from Changsha Tianqin Biotechnology Co., Ltd. (Hunan, China) (qualification certificate No: SCXK (Xiang) 2022-0011). The mice were kept in an aerated cage with an ambient temperature of 25°C ± 2°C, relative humidity of (55 ± 5) %, and alternating white light/darkness for 12 h/12 h, and fed a standard diet and water. The animal experiment was approved by the Animal Experiment Ethics Committee of Guizhou University of Chinese Medicine (Approval No. 20230039).

After 1 week of adaptive feeding, the mice were randomly divided into the following four groups: the blank control group (CG), and low, medium, and high-dose CTD groups (n = 10 mice per group). CTD model mice were given 0.5, 1.0, and 1.5 mg/kg CTD solution, and the control group was given equal volume CMC-Na solution to establish the CTD-induced liver injury model. After 14 days of intragastric administration (once a day), the mice in each group were weighed and anesthetized with 0.5% sodium solution of pentobarbital, before collecting their orbital blood. The blood was left at room temperature for 30 min, before centrifuging at 3,000 g at 4°C for 10 min, and collecting the serum for later use. The animals were sacrificed by cervical dislocation and dissected under an ice bath, and the liver tissues were quickly separated, weighed, and recorded. Part of the liver tissues were fixed in 4% paraformaldehyde and 2.5% glutaraldehyde solutions, and the remaining samples were first placed in liquid nitrogen and then transferred to −80°C for later use.

### 2.2 Liver index

The mouse liver index was calculated as = wet weight of liver (g)/last body weight (g) × 100%.

### 2.3 Detection of serum alanine aminotransferase (ALT) and aspartate aminotransferase (AST) levels

The activity of ALT and AST enzymes in mouse serum was detected using kits purchased from Nanjing Jiancheng Biotechnology Research Institute (Jiangsu, China). according to the manufacturer’s instructions.

### 2.4 Pathological observation and immunohistochemical staining of the liver tissue

The liver tissues fixed in paraformaldehyde for 24 h were dehydrated by gradient ethanol, transparent by xylene, embedded in paraffin, and then cut into 4-μm thick sections, before staining with hematoxylin–eosin (HE), fixing with neutral gum tablets, and storing at room temperature. Histopathological observations were made under an optical microscope, followed by photographing.

Immunohistochemical staining was performed using the kit (Sangon Biotech, Shanghai, China), and the images were observed under an optical microscope. ImageJ (Java 1.8.0 by Wayne Rasband, National Institutes of Health, Bethesda, MD, USA) was used to quantify the positive expression rate of the DDIT4 protein (three slices were randomly selected for each animal, with 18 slides per group).

### 2.5 Cell culture

Cantharidin, 3-methyladenine (MCE, USA), and rapamycin (MCE, USA) were prepared into 100, 50, and 20 mM solutions with DMSO, respectively, and stored at −20°C for future use. Upon use, the corresponding culture medium was diluted to the required concentration. Human normal liver cells (L-O2) were purchased from Saibaikang (Shanghai) Biotechnology Co., Ltd. and were identified by STR (iCell-h054). After rehydration and passage, L-O2 cells were expanded and cultured under 37°C and 5% CO_2_ saturation humidity.

### 2.6 Cell viability assay

L-O2 cells in the logarithmic growth stage and a good growth state were used for experiments. Briefly, 5 × 10^3^ cells/well were inserted into a 96-well plate, and a blank group was set up and cultured overnight at 37°C. According to the different groups and cell treatment settings, the cells were treated separately and cultured in an incubator at 37°C and 5% CO_2_. The groups were as follows: 1) normal cells; 2) 0.9375 μmol/L CTD group; 3) 1.875 μmol/L CTD group; 4) 3.75 µmol/L CTD group; 5) 7.5 µmol/L CTD group; 6) 15 µmol/L CTD group; 7) 30 μmol/L CTD group; and 8) 60 μmol/L CTD group. L-O2 cells were treated with different concentrations of CTD for 6, 12, 24, and 36 h. After the specified time had elapsed, 10 μL CCK-8 reagent was added to each well (Elabscience, China) and cultured at 37°C for 4 h. The OD_450_ in each well was determined by enzyme-labeled assay.

### 2.7 qRT–PCR

After extracting total RNA from liver tissues (0.5, 1.0, and 1.5 mg/kg CTD exposure groups) or L-O2 cells (0, 7.5, 15, and 30 μmol/L CTD treatment for 24 h) using TRIzol (Ambion, USA), HiScript ^®^ II Q RT SuperMix for qPCR (+gDNA wiper) (Vazyme, China) was reverse transcribed into cDNA. According to the instructions of the SYBR Green Master Mix (Vazyme, China) assay kit, GAPDH was used as the internal reference gene to detect the relative DDIT4 mRNA expression level. Primer Premier 5.0 (Canada) was used to design the primer sequences, as shown in Supplementary Table S1. The primers were synthesized by Beijing Qingke Biotechnology Co., Ltd. (China). The final data were quantitatively analyzed using the 2^-△△Ct^ method.

### 2.8 Cell transfection with siRNA

To knock down DDIT4, L-O2 cells were transfected with double-stranded siRNA (GenePharma, Shanghai, China). The sequence of SiDDIT4 was as follows: sense, GUU​UGU​GUA​UCU​UAC​UGG​UTT; antisense, ACC​AGU​AAG​AUA​CAC​AAA​CTT. L-O2 cells were inoculated in 6-well plates, cultured in normal medium for 24 h, and then replaced with serum-free RPMI 1640 2 h before transfection. Transfection was performed according to the following experimental groups: 1) normal group; 2) interference negative control (siNC) group; 3) siDDIT4 group; 4) siDDIT4+Rap group; 5) siDDIT4+3-MA group; 6) CTD + siNC group; 7) CTD + si DDIT4 group; 8) CTD + siDDIT4+Rap group; and 9) CTD + siDDIT4+3-MA group. Following the manufacturer’s instructions, Lipofectamine 3,000 (Mei5 Biotechnology, Beijing, China) was used to transfect SiDDIT4 or siNC directly into target cells. According to the group, CTD was added for 12 h, and then Rap or 3-MA was added for 12 h.

### 2.9 Western blotting

RIPA protein lysis solution (Beyotime, China) was added to the processed mouse liver tissue or L-O2 cells, before placing them on ice for thorough grinding and lysis. Subsequently, the samples were centrifuged at 13,400 *g* at 4°C for 5 min, before collecting the supernatant and measuring the protein concentration using the BCA protein concentration assay kit (Beyotime, China). The tissue protein samples were subjected to SDS-PAGE gel electrophoresis and membrane transfer, before sealing with 5% skimmed milk powder for 2 h. The following antibodies were dripped: DDIT4 (1:1000, A14135, Abclonal, China), Beclin-1 (1:1000, A7353, Abclonal, China), LC3 (1:1000, AF5402, Affinity, USA), mToR (1:1000, AF6308, Affinity, USA), p-mToR (1:1000, AF3308, Affinity, USA), Bax (1:1000, AF0120, Affinity, USA A), Bcl-2 (1:1000, AF6139, Affinity, USA) β-Actin (1:1000, BM0627, Bosterbio, China), and GAPDH (1:1000, AB-P-R 001, Goodhere, China), where β-Actin and GAPDH were used as internal references. The antibodies were incubated overnight at 4°C, and then hybridized with HRP-labeled goat anti-rabbit IgG secondary antibody (1:10,000, BA1054, Bosterbio, China). Excess antibody was washed off with TBST, and ECL chemiluminescence was used for development exposure. The film was dried and scanned, and the grayscale value of the film was analyzed and photographed using Image Pro Plus.

### 2.10 Flow cytometry

L-02 cells in each group were inoculated into 6-well plates and incubated at 37°C overnight, before discarding the medium and washing the adherent cells twice with phosphate buffered saline (PBS). To obtain the suspension, the cells were digested with 0.25% trypsin without EDTA, transferred to a new EP tube, centrifuged at 1000 *g* at 4°C for 5 min, the supernatant was discarded, and the cells were rinsed twice with PBS at 1000 g for 5 min. Subsequently, apoptosis was detected using the AnnexinV-APC/7-AAD apoptosis detection kit (Keygen Biotech, KGA1026, China), and on-machine analysis was performed by flow cytometry (Beckman Coulter, cytoFLEX, USA).

### 2.11 Transmission electron microscopy

Small pieces of liver tissue or L-O2 cells were fixed with 2.5% glutaraldehyde. The steps of cleaning, fixation, dehydration, polymerization, continuous ultra-thin sectioning, and staining followed the method described in our previous publication (Tang et al., 2018). Transmission electron microscopy (Hitachi, ht7800/ht7700, Japan) was used to obtain images at 100 kV.

### 2.12 Hoechst 33,342 staining

siNC- or SIDDIT4-transfected L-O2 cells were transfected with the GFP-LC3 plasmid (provided by Hanshenti Technology, Inc.) using Lipofectamine 2000 reagent (Invitrogen, USA). L-O2 cells were inoculated on a 24-well plate and allowed to attach. Subsequently, the cells were soaked with PBS three times, for 3 min each time, before fixing with 4% paraformaldehyde drops for 15 min. The slides were soaked with PBS three times, for 3 min each time, before using absorbent paper to blot the PBS, and adding Hoechst 33,342 dye solution to the slides (Beyotime, China). After incubation at room temperature for 5 min, the slides were soaked with PBS three times, for 3 min each time, the liquid on the slides was dried with absorbent paper, and the slides were sealed with a sealing solution containing the anti-fluorescence quench agent Southernbiotech (USA). The images were observed and collected under a confocal laser microscope (Nikon C2, Japan).

### 2.13 Statistical analysis

All data were statistically analyzed using SPSS 28.0 (SPSS Inc., Chicago, IL). Dunnett’s *t*-test was used to compare the multiple groups, and one-way analysis of variance (ANOVA) was used to analyze the statistical differences among the multiple groups. The values are expressed as the mean ± standard deviation, and *p*-values < 0.05 were considered to indicate statistical significance.

## 3 Results

### 3.1 CTD-induced liver injury in mice

**FIGURE 1:**
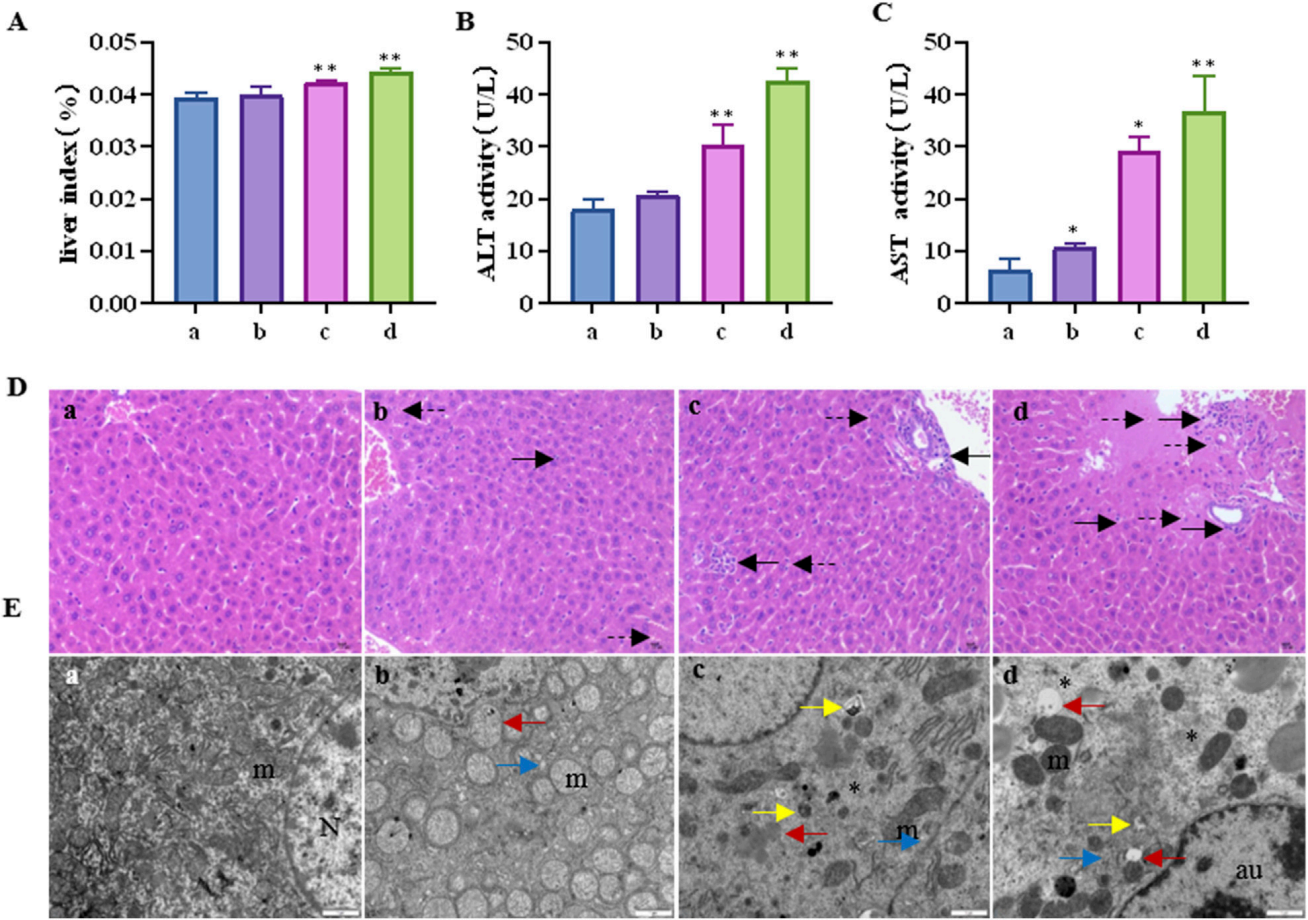
Liver injury induced by cantharidin in mice. **(A)** Mouse liver index; **(B)** Serum AST activity; **(C)** Serum ALT activity; **(D)** Pathological diagram of liver tissue (H&E), in which the solid arrows indicate spotty necrosis of liver cells, and the dotted arrows indicate inflammatory cell infiltration; **(E)** Transmission electron microscope image of liver tissue, the red arrow indicates mitochondrial swelling and ridge disappearance, blue arrow indicates endoplasmic reticulum expansion, yellow arrow indicates mitochondrial autophagy, the asterisk indicates reduced organelles in the cytoplasm and vacuolation, N: nucleus, m: mitochondria, au: Autophagolysosome (* indicates significant difference compared with control group, * *p* < 0.05, ** *p* < 0.01, **(a)** control group, **(b)** 0.5 mg/kg CTD, **(c)** 1 mg/kg CTD, **(d)** 1.5 mg/kg CTD, Figures 2, 3 are the same).

The liver index of the mice is shown in Figure 1A. Compared to the control group, the liver index showed a tendency to increase with increasing CTD exposure concentration, especially in the 1 mg/kg and 1.5 mg/kg CTD exposure groups (*p* < 0.05). The serum liver function indices (AST and ALT activities) are shown in Figures 1B, C. Compared to the control group, AST activity was significantly increased. In the low, medium, and high concentrations, CTD groups were increased by 1.67 (*p* > 0.05), 4.55 (*p* < 0.01), and 5.74 (*p* < 0.01) times, respectively. ALT activity was increased by 1.16 (*p* > 0.05), 1.69 (*p* < 0.01), and 2.38 (*p* < 0.01) times. Histopathological examination of the livers of mice showed spotty necrosis and inflammatory cell infiltration (Figure 1D). Ultrafine structure observation showed that after CTD exposure, mitochondrial swelling and ridge disappearance, endoplasmic reticulum expansion, mitochondrial autophagosomes, and lysosomes appeared in the cytoplasm, and even reduced organelles and cellular vacuolation in the cytoplasm at high concentrations (Figure 1E). The pathological damage became more severe with the increase in CTD exposure concentration.

### 3.2 CTD leads to increased DDIT4 mRNA and protein expression levels in mouse liver tissue

**FIGURE 2:**
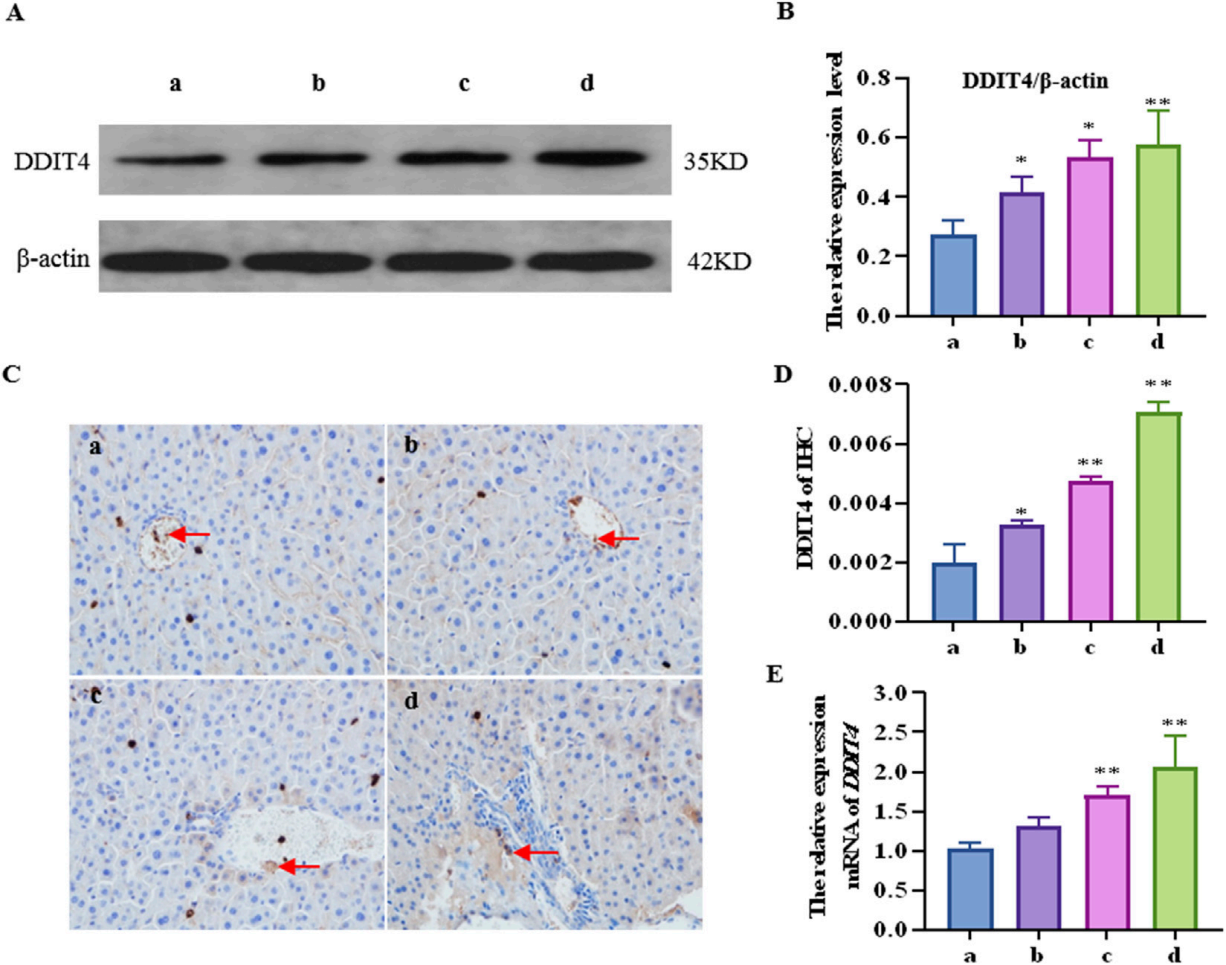
Effects of cantharidin on mRNA and protein expression of DDIT4 in mouse liver. **(A)** western blot gel electrophoresis of DDIT4, with β-actin as the internal reference; **(B)** Immunohistochemical map of DDIT4 protein, the brown area indicated by the arrow represents the positive rate; **(C)** Quantitative protein map by western blot; **(D)** Immunohistochemical quantitative map; **(E)** qRT-PCR diagram of DDIT4 gene, with β-actin as the internal reference gene.

The results of western blotting and immunohistochemistry (Figures 2A–D) showed that the DDIT4 protein expression levels in all concentration groups were significantly increased after CTD exposure (*p* < 0.05), particularly at high concentrations (*p* < 0.01). Moreover, the qRT–PCR results showed that compared to the control group, the mRNA relative expression level of the *DDIT4* gene in the medium- and high-concentration CTD-exposed groups was significantly increased (*p* < 0.01) (Figure 2E).

### 3.3 CTD causes increased autophagy and apoptosis levels in the mouse liver

**FIGURE 3:**
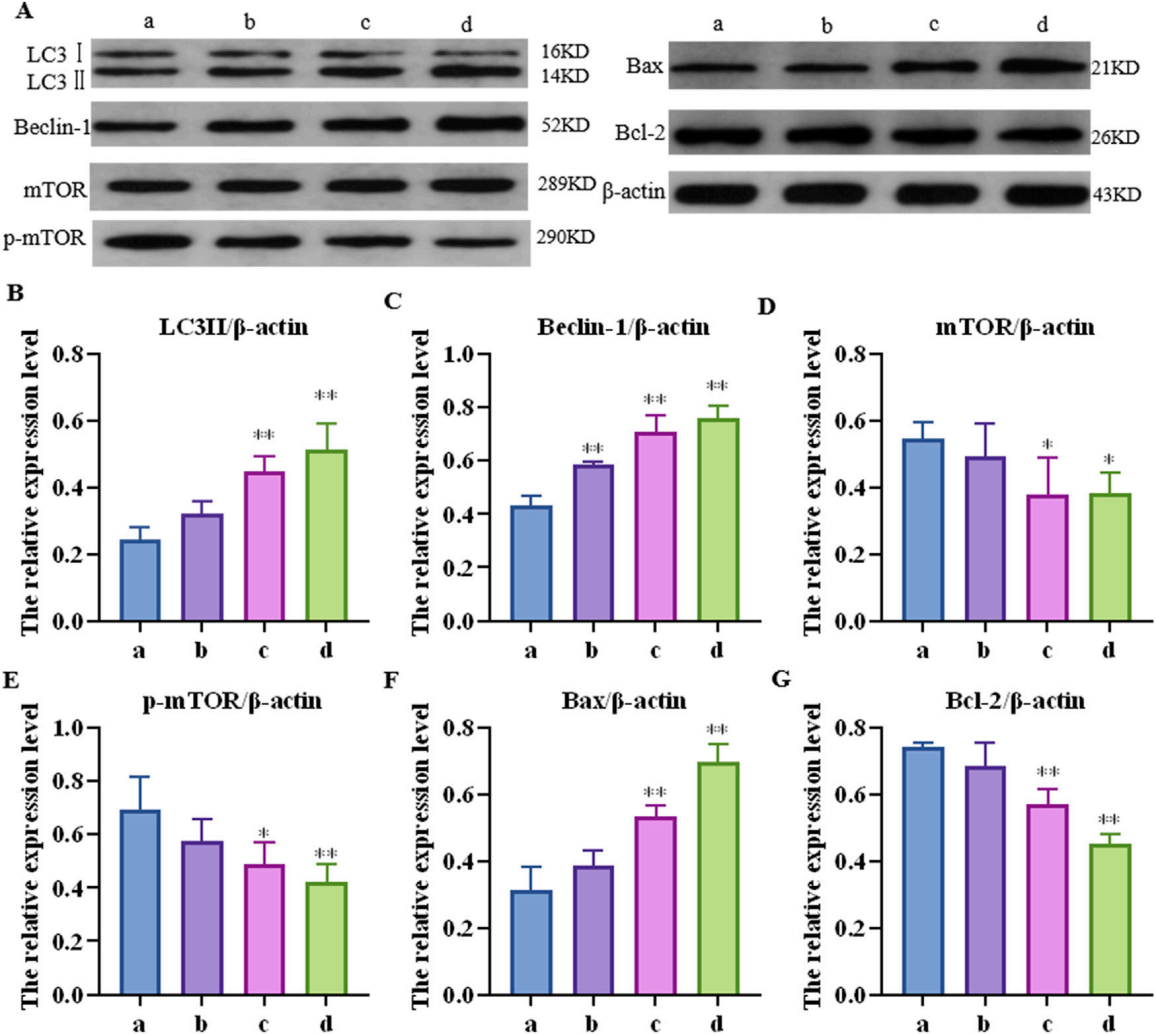
Effects of cantharidin on the expression of autophagy and apoptosis-related proteins in mouse liver. **(A)** western blot gel electrophoresis; **(B–G)**: represents the relative expression of LC3Ⅱ, Beclin-1, mTOR, p-mTOR, Bax and Bcl-2, respectively.

Further detection results of the mTOR pathway, autophagy-related proteins (LC3Ⅱ and Beclin-1), and apoptosis key proteins (Bax and Bcl-2) in the mouse liver are shown in Figure 3. Compared to the control group, the relative protein expression of LC3-II in each concentration group was upregulated by 1.33 (*p* > 0.05), 1.84 (*p* < 0.05), and 2.10 (*p* < 0.01) times, respectively; Beclin-1 was upregulated 1.35 (*p* < 0.01), 1.84 (*p* < 0.01), and 2.10 (*p* < 0.01) times, respectively; mTOR was downregulated 1.31 (*p* > 0.05), 1.44 (*p* < 0.05), and 1.44 (*p* < 0.01) times, respectively; p-mTOR was downregulated 1.13 (*p* > 0.05), 1.35 (*p* < 0.05), and 1.60 (*p* < 0.01) times, respectively; Bax was increased by 1.23 (*p* > 0.05), 1.71 (*p* < 0.01), and 2.23 (*p* < 0.01) times, respectively; and Bcl-2 was downregulated by 1.08 (*p* > 0.05), 1.30 (*p* < 0.01), and 1.65 (*p* < 0.01) times, respectively.

### 3.4 CTD exhibited toxicity to L-O2 cells and increased DDIT4 expression

**FIGURE 4:**
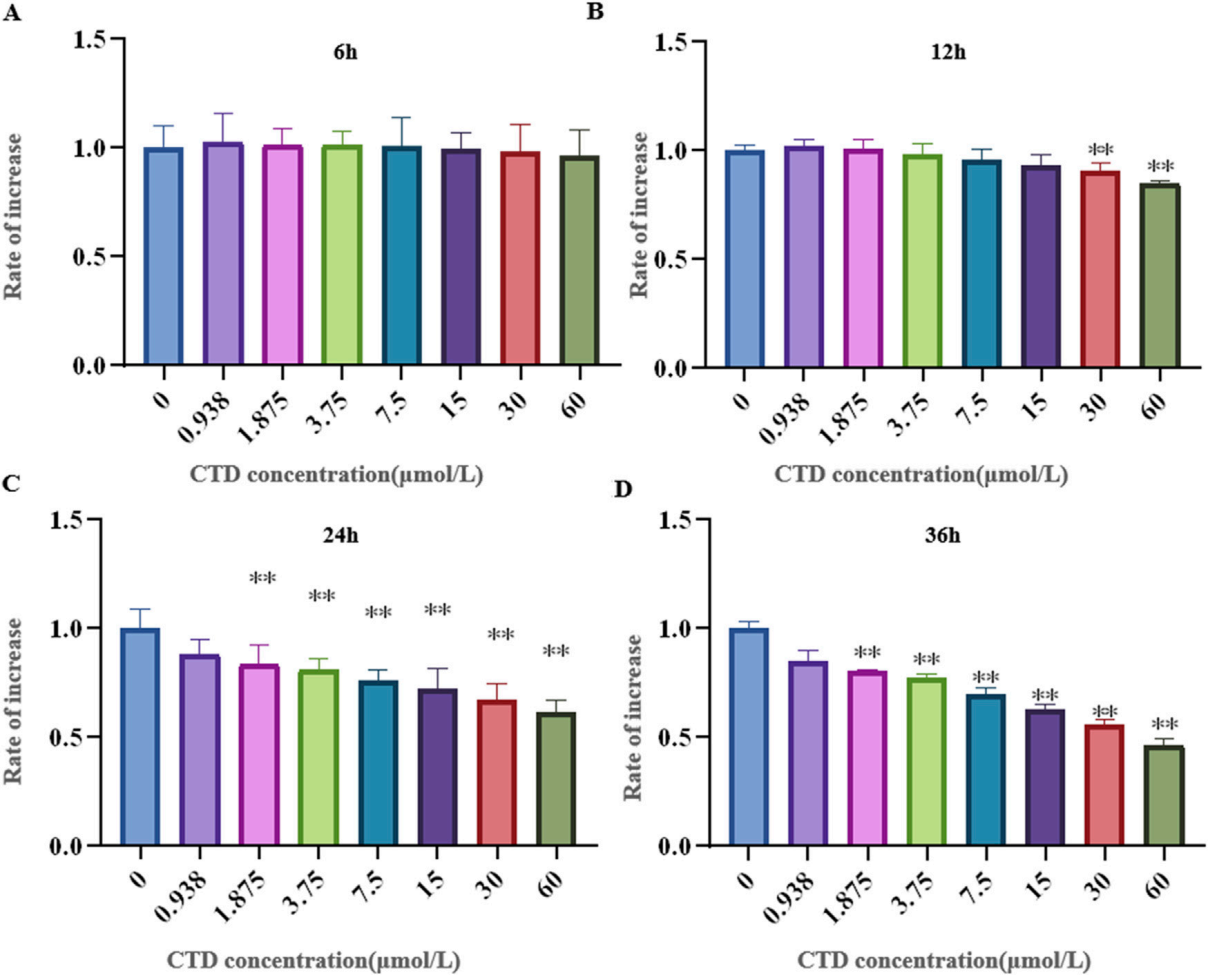
Effects of CTD on L-O2 cytotoxicity. **(A–D)**: CCK-8 detected the activity of L-02 cells after CTD exposure at different times, and a-h represented 0, 0.9375, 1.875, 3.75, 7.5, 15, 30 and 60 μmol/L CTD treatment groups, respectively. (* indicated a significant difference compared with the control group, * *p* < 0.05, ** *p* < 0.01, same in Figures 5, 6)

**FIGURE 5:**
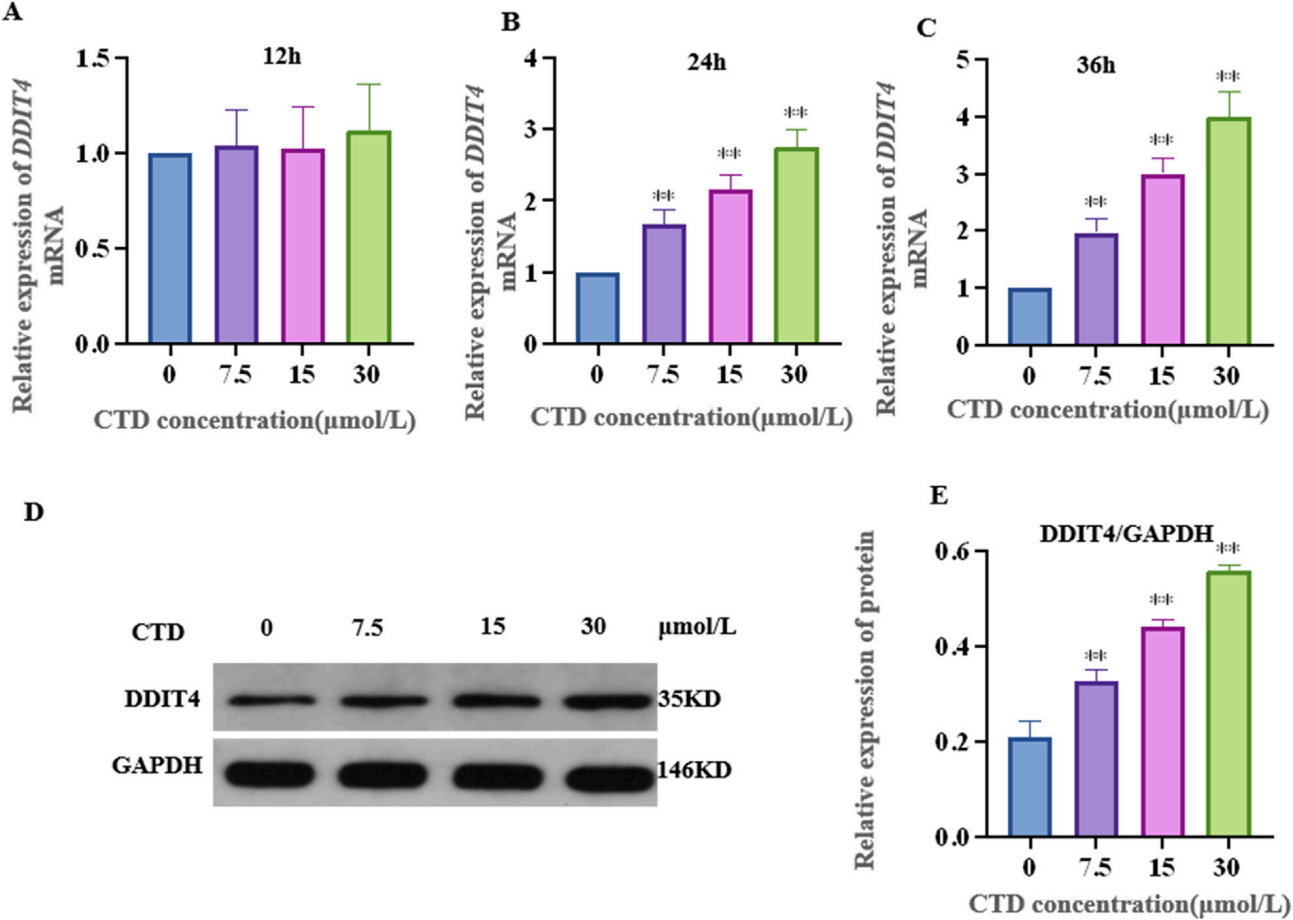
Effects of CTD on DDIT4 expression. **(A–C)**: The mRNA relative expression level of the DDIT4 gene in L-O2 cells after 12, 24, and 36 h of CTD exposure was detected by qRT-PCR, and GAPDH was the internal reference gene. **(D, E)**: WB detected the relative expression level of DDIT4 protein in L-O2 cells after 24 h exposure to CTD.

The results of the CCK-8 assay and L-O2 cell viability experiments are shown in Figure 4. Compared to the control group, after 6 h exposure to CTD, the cell viability of each concentration group showed no significant change (*p* > 0.05). After 12 h, with the exception of the 30 and 60 μmol/L CTD exposure groups, the cell viability decreased significantly (*p* < 0.01), while no significant changes were observed in the other groups (*p* > 0.05). After 24 and 36 h, with the exception of the 0.9375 μmol/L CTD exposure group, the cell viability was significantly decreased in all other groups (*p* < 0.01). According to the results of CCK-8, we detected the relative DDIT4 gene mRNA expression in L-O2 cells after exposure to CTD (15, 30, and 60 μmol/L) (12, 24 and 36 h) by qRT–PCR. The results showed that, compared to the control group, after exposure to CTD for 12 h, there was no significant change in the DDIT4 gene mRNA expression level in all concentration groups (*p* > 0.05) (Figure 5A). Moreover, after 24 and 36 h, the mRNA expression level of the DDIT4 gene in all concentration groups was significantly increased (*p* < 0.01) (Figures 5B, C). We further determined the relative expression level of DDIT4 protein after 24 h exposure to CTD, and the results showed that the DDIT4 protein expression in each concentration group was significantly increased compared to that in the control group (*p* < 0.01) (Figures 5D, E).

### 3.5 CTD increased autophagy and apoptosis of L-O2 cells

**FIGURE 6:**
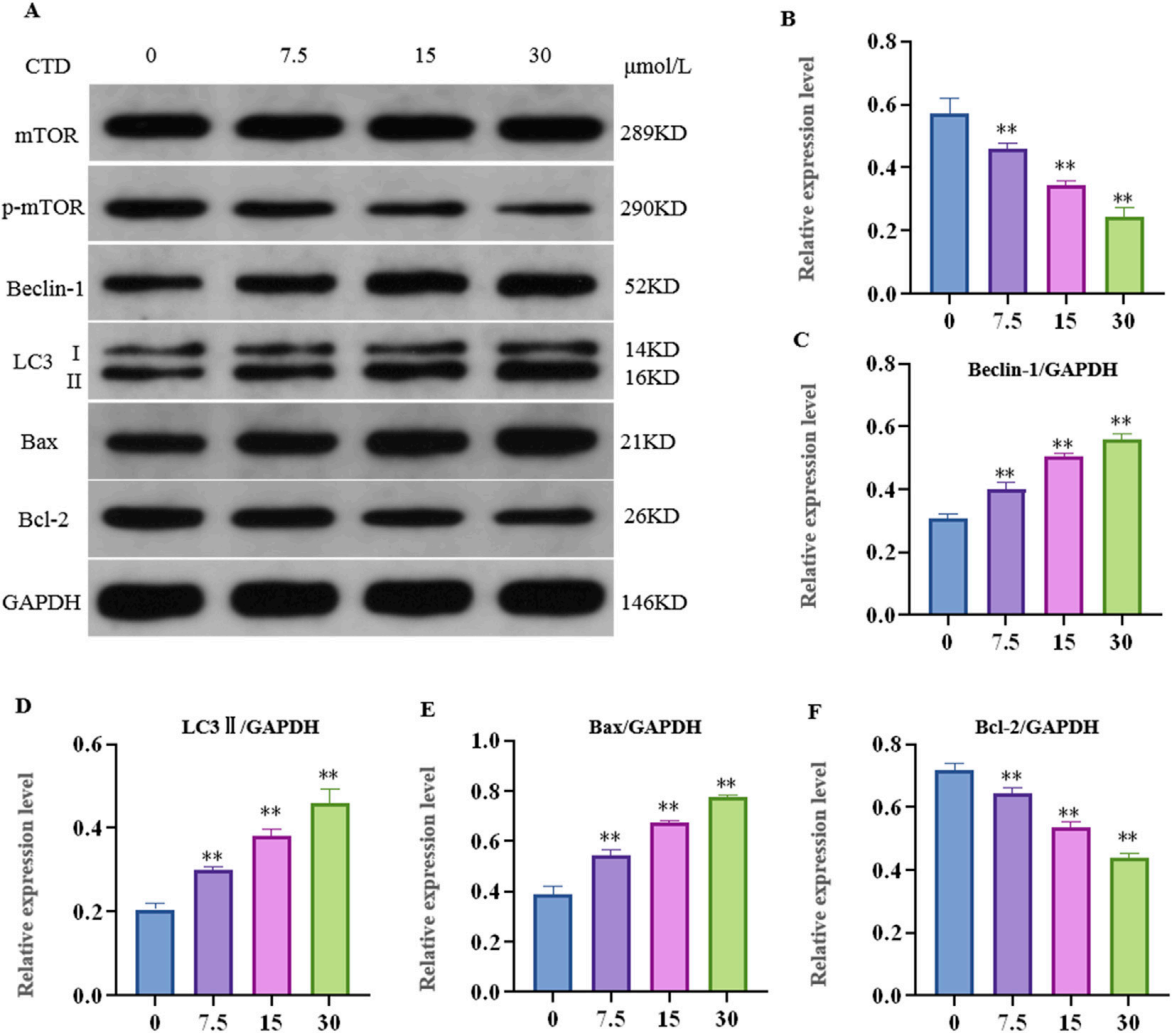
Effects of cantharidin on autophagy and apoptosis-related protein expression in L-O2 cells. **(A)** western blot gel electrophoresis; **(B–F)**: indicates the relative protein expression of p-mTOR, LC3Ⅱ, Beclin-1, Bax, and Bcl-2, respectively.

After 24 h of CTD exposure, the results of the experiments pertaining to the mTOR pathway, autophagy, and apoptosis-related proteins in L-O2 cells are shown in Figure 6. Compared to the control group, the relative expression levels of p-mTOR and Bcl-2 proteins in the 7.5, 15 and 30 μmol/L CTD exposure groups were significantly decreased (*p* < 0.01), while those of LC3Ⅱ, Beclin-1, and Bax were significantly increased (*p* < 0.01).

### 3.6 Effects of DDIT4 knockdown and autophagy activation/inhibition on autophagy and L-O2 cell apoptosis

RNAi was used to knock down the transcription and translation of the DDIT4 gene in cells, and western blotting was used to detect the expression of the DDIT4 protein. The results showed that the interference sequence targeting L-O2 cells could effectively knock down the expression of DDIT4 (*p* < 0.01). We selected the sequence with the highest knockdown for subsequent experiments (Supplementary Figure S1).

#### 3.6.1 Western blot to detect autophagy and apoptosis of L-O2 cells

**FIGURE 7:**
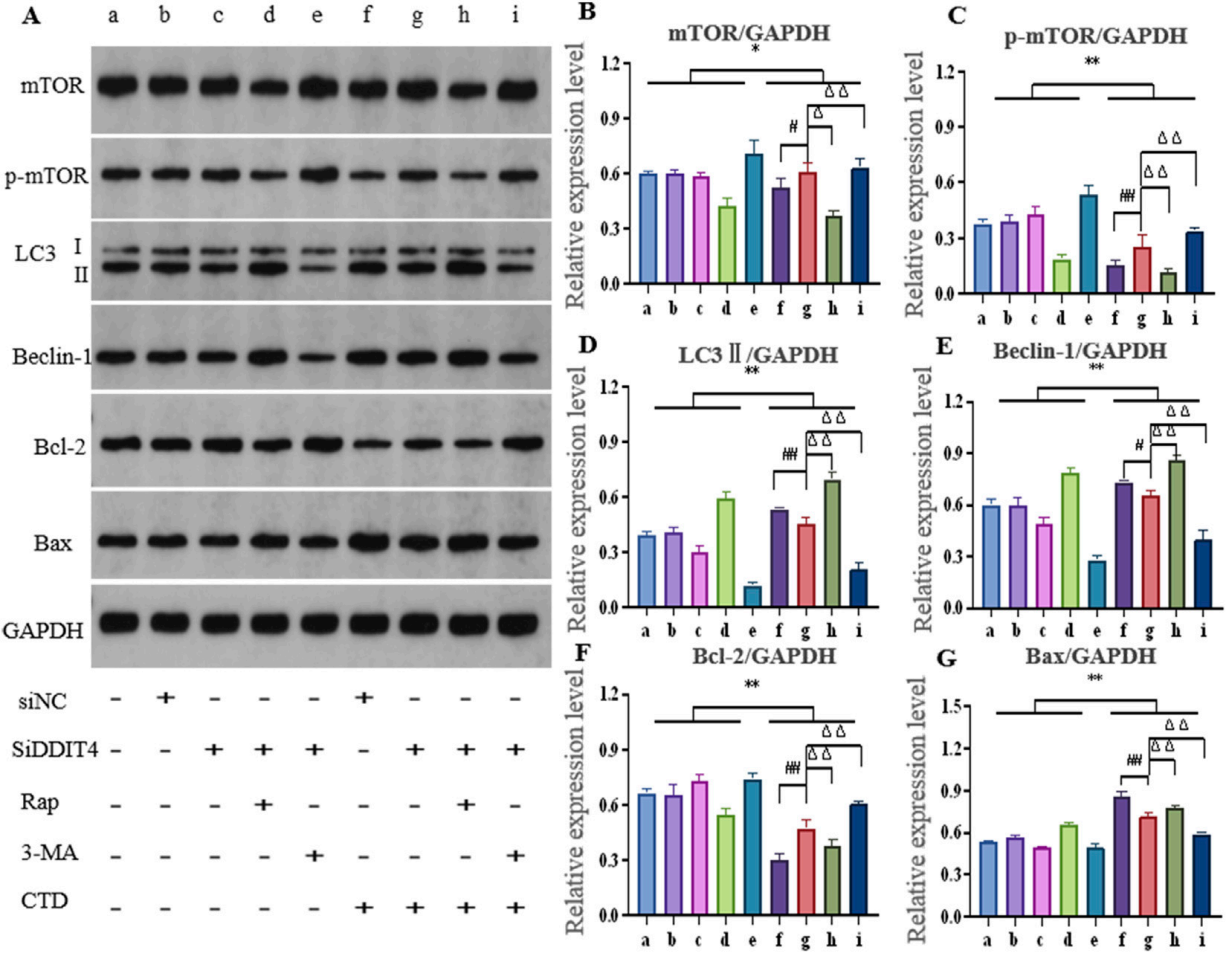
Effects of cantharidin on autophagy and apoptosis-related protein expression in L-O2 cells. **(A)** western blot gel electrophoresis; **(B–G)**: Histogram of protein relative expression of mTOR, p-mTOR, LC3Ⅱ, Beclin-1, Bcl-2 and Bax, respectively (Control: b, c, d, e; CTD: f, g, h, I; * for vs. Control, * *p* < 0.05, ** *p* < 0.01; # indicates vs. CTD + SiNC, ## *p* < 0.01; Δ represents vs. CTD + SiDDIT4, ΔΔ *p* < 0.01, same as Figure 8).

After knocking down DDIT4 and using the autophagy activator Rap or inhibitor 3-MA, the expression of autophagy- and apoptosis-related proteins in L-O2 cells is shown in Figure 7. The relative expression levels of mTOR (*p* < 0.05), p-mTOR (*p* < 0.01), and Bcl-2 (*p* < 0.01) in the CTD-exposed groups were significantly decreased, whereas those of LC3-II, Beclin-1, and Bax were significantly increased (*p* < 0.01). After DDIT4 deletion, compared to the CTD + SiNC group, the relative expression levels of mTOR (*p* < 0.05), p-mTOR (*p* < 0.01), and Bcl-2 (*p* < 0.01) in the CTD + SiDDIT4 group were significantly increased, while those of LC3-II (*p* < 0.01), Beclin-1 (*p* < 0.05), and Bax (*p* < 0.01) were significantly decreased. After treatment with Rap, compared to the CTD + SiDDIT4 group, the relative expressions of mTOR, p-mTOR, and Bcl-2 proteins in the CTD + SiDDIT4+Rap group were significantly decreased (*p* < 0.01), whereas those of LC3-II, Beclin-1, and Bax were significantly increased (*p* < 0.01). After 3-MA treatment, compared to the CTD + SiDDIT4 group, the relative expressions of p-mTOR and Bcl-2 proteins in the CTD + SiDDIT4+ 3-MA group were significantly increased (*p* < 0.01), whereas LC3-II, Beclin-1, and Bax were significantly decreased (*p* < 0.01).

#### 3.6.2 Autophagy of L-O2 cells detected by confocal microscopy

**FIGURE 8:**
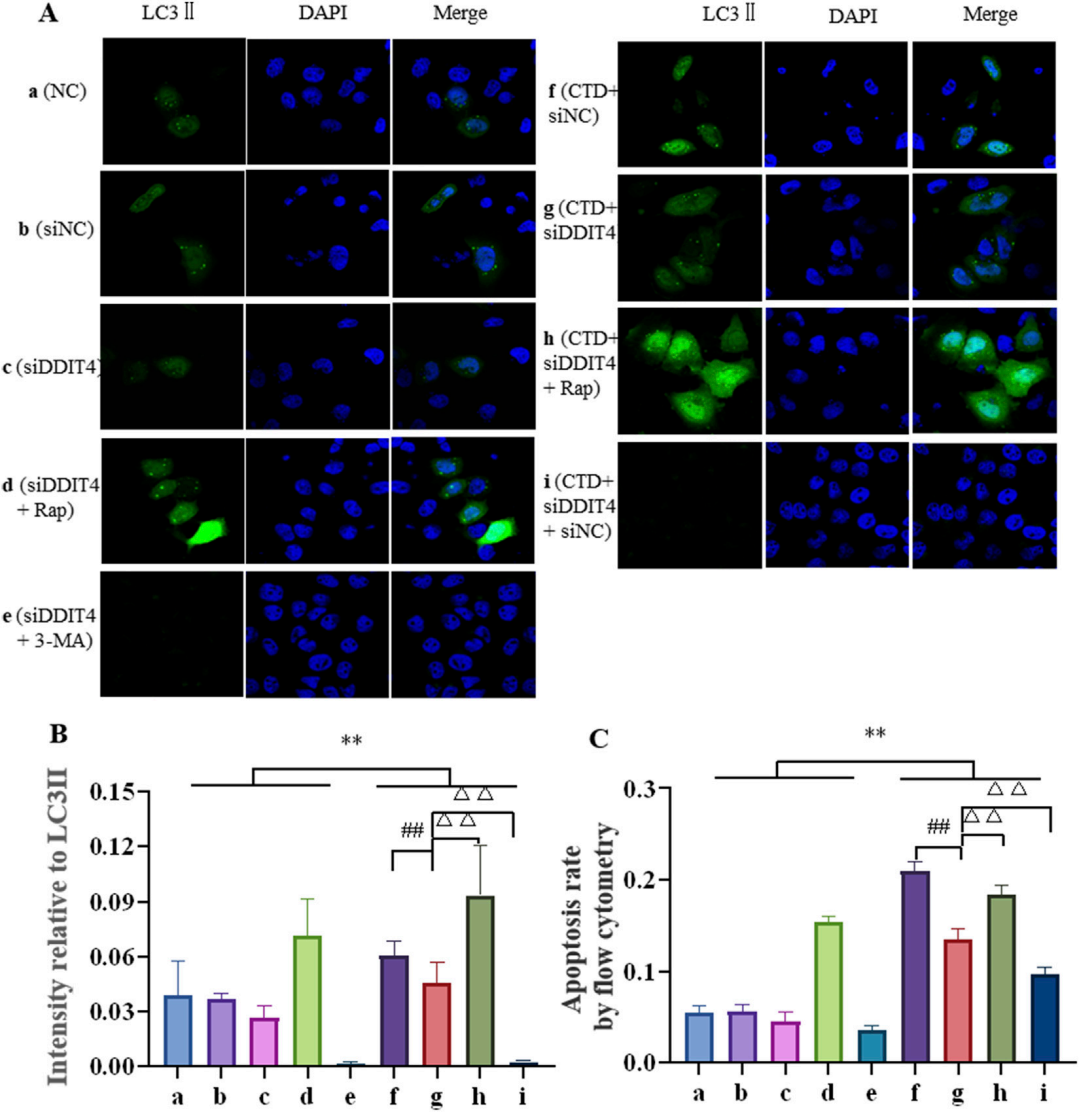
Hoechst33342 staining of LC3Ⅱ **(A, B)**, and the apoptosis level of L-O2 cells was detected by flow cytometry **(C)**.

The expression level of LC3-II in L-O2 cells was further detected by Hoechst 33,342 staining, with the results shown in Figures 8A, B. Compared to the control groups, LC3-II expression in the CTD-exposed groups was significantly increased (*p* < 0.01). After DDIT4 deletion, LC3Ⅱ expression in the CTD + SiDDIT4 group was significantly decreased compared to that in the CTD + SiNC group (*p* < 0.01). After treatment with Rap, LC3-II expression in the CTD + SiDDIT4+Rap group was significantly higher than that in the CTD + SiDDIT4+Rap group (*p* < 0.01). After 3-MA treatment, LC3-II expression in the CTD + SiDDIT4+3-MA group, LC3Ⅱ expression in the CTD + SiDDIT4+3-MA group was significantly decreased (*p* < 0.01).

#### 3.6.3 Flow cytometric detection of L-O2 cell apoptosis

Flow cytometry was further used to detect L-O2 cell apoptosis, with the results shown in Figure 8C and Supplementary Figure S2. Compared to the control groups, the apoptosis rate of the CTD-exposed groups was significantly increased (*p* < 0.01). After DDIT4 knockdown, compared to the CTD + SiNC group, the apoptosis rate of the CTD + SiDDIT4 group was significantly decreased (*p* < 0.01). Compared to the CTD + SiDDIT4+Rap group, the apoptosis rate of the CTD + SiDDIT4+Rap group was significantly increased (*p* < 0.01). After 3-MA treatment, compared to the CTD + SiDDIT4 group, the apoptosis rate of the CTD + SiDDIT4+3-MA group was significantly decreased (*p* < 0.01).

#### 3.6.4 Transmission electron microscopy observation of L-O2 cell autophagy

**FIGURE 9:**
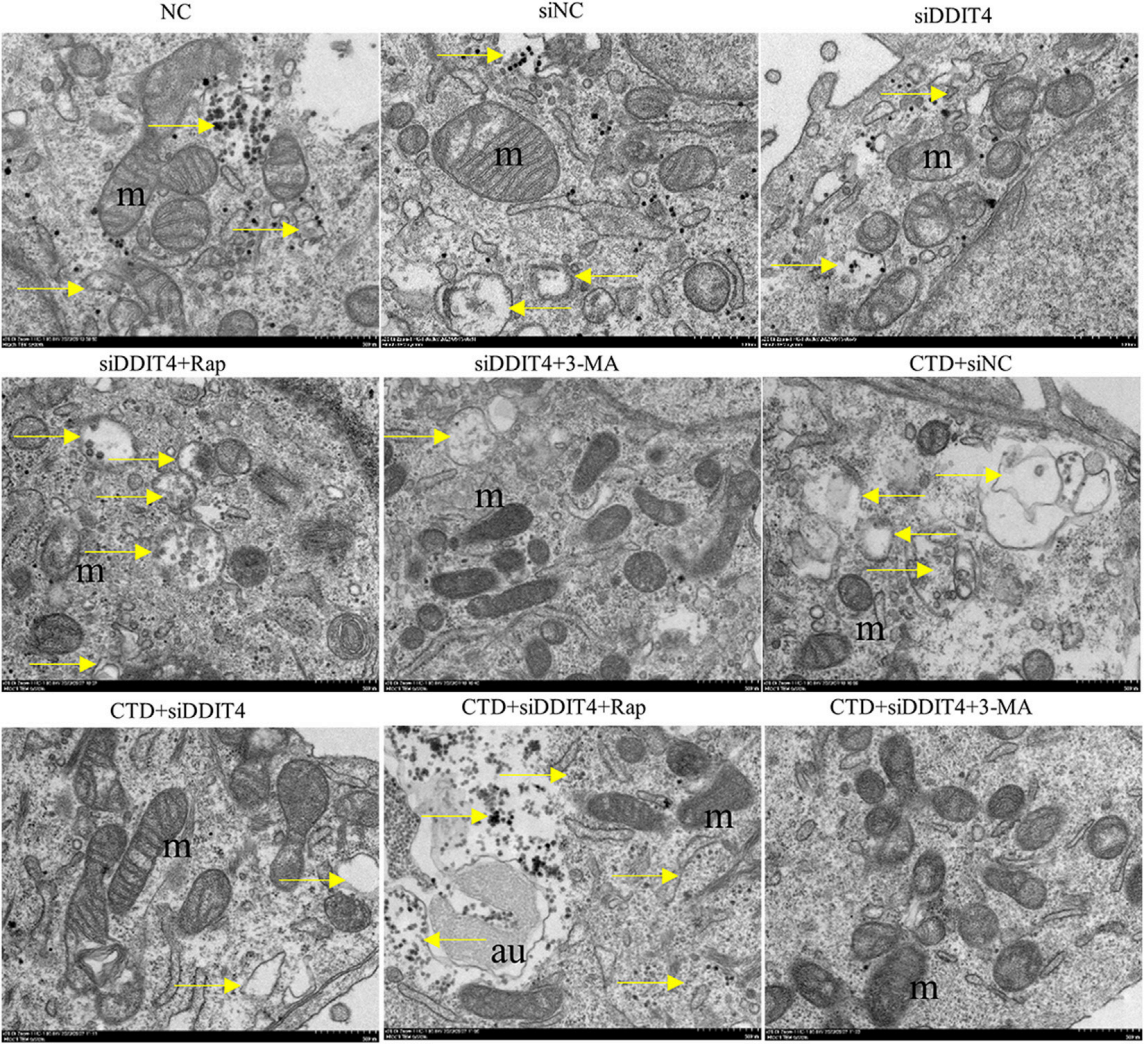
Transmission electron microscopy of L-O2 cells. The yellow arrows in the figure indicate mitochondrial autophagy, m: mitochondria, au: autophagy lysosomes.

In each group, autophagy was observed by transmission electron microscopy, with the results shown in Figure 9. Mitochondrial autophagy was observed in the NC group and all control groups. After CTD exposure, the mitochondrial autophagy levels increased to a certain extent, whereas Rap treatment significantly increased mitochondrial autophagy. In contrast, mitochondrial autophagy was significantly inhibited after 3-MA treatment. The observed mitochondrial autophagy results are consistent with the western blotting and immunofluorescence results.

## 4 Discussion

Due to the strong killing effect of its main active ingredient CTD on various stubborn tumors, the traditional Chinese medicine Mylabris has been developed into numerous anti-tumor traditional Chinese medicines for the market, including Aidi Injection, Sodium Cantharidinate Vitamin B6 Injection, and Compound Cantharidin Capsules (Liu et al., 2020b; He et al., 2021). However, the therapeutic dose of CTD is very close to its toxic dose, and long-term use can lead to serious liver and kidney toxicity (Jin et al., 2023). A recent review summarized the current mechanisms of CTD-induced hepatotoxicity, indicating that CTD leads to varying degrees of liver damage by activating endogenous and exogenous pathways, resulting in hepatocyte apoptosis and autophagy (Jin et al., 2023). However, the mechanism of CTD-induced hepatotoxicity has not been fully elucidated. In this study, we investigated the important role of the DDIT4/mTOR signaling pathway in CTD-induced autophagy and apoptosis of liver cells, providing an important theoretical basis for a better understanding of the mechanism of CTD-induced hepatotoxicity.

It is well known that an increase in liver index and serum AST and ALT levels are important indicators for the evaluation of liver injury. In our study, the liver index and serum ALT and AST levels of mice increased significantly after exposure to different concentrations of CTD and showed dose dependence. Moreover, obvious pathological damage of mouse liver tissue was observed by HE staining and transmission electron microscopy, suggesting that CTD can cause liver injury in mice, confirming successful generation of the animal model. Through transmission electron microscopy, we also observed that liver cell damage is closely related to mitochondrial autophagy. Previous studies have shown that CTD can induce autophagy and apoptosis in L-O2 cells (Liu et al., 2020a). We further detected autophagy-related proteins (Beclin-1 and LC3-II) and apoptosis-related proteins (Bax and Bcl-2) in the mouse liver. Beclin-1, also known as the autophagy-related gene (Atg) 6, is a recognized autophagy regulator and a key component in inducing autophagosome formation and lysosomal biogenesis (Fekadu and Rami, 2016). LC3-II is an important marker molecule of autophagosomes. During autophagy, LC3-I is modified and processed by ubiquitin-like systems, including Atg7 and Atg3, producing LC3-II, with a molecular weight of 14 kDa. The LC3-II content is directly proportional to the degree of autophagy (Galluzzi and Green, 2019), and enhanced expression of Beclin-1 and LC3-II is necessary for autophagy (Wang, 2008). In our study, with an increase in the CTD exposure concentration, both Beclin-1 and LC3-II showed significant increases, indicating that CTD can induce autophagy in the mouse liver. Related studies have shown that the activation of Bax is a key event in the occurrence of apoptosis (Liu et al., 2011), whereas BCL2 can exert anti-apoptotic effects by inhibiting the release of cytochrome C and apoptosis initiation factors (Schultz and Harrington, 2003). Our results revealed that the expression of Bax protein was increased and that the expression of Bcl-2 was inhibited after CTD exposure, indicating that CTD caused apoptosis in the mouse liver. Our results are consistent with those of Liu et al. (2020a), who reported that CTD can induce autophagy and apoptosis of L-O2 cells *in vitro*.

mTOR is the main regulator of cell growth, proliferation, and metabolism, as well as being an inhibitor of autophagy induction (Li et al., 2012). Research has shown that mTOR signaling is significantly inhibited in liver injury induced by ischemia-reperfusion, carbon tetrachloride (CCl_4_), and acetaminophen (Rao et al., 2017; Gao et al., 2020; Fang et al., 2023). In our study, both mTOR and p-mTOR were significantly downregulated in the mouse liver after CTD exposure, indicating that CTD could inhibit the mTOR pathway. It is well known that inhibition of the mTOR pathway promotes autophagy, while related studies have shown that inhibition of mTOR also promotes apoptosis (Wang et al., 2022; Xie et al., 2023; Kumari et al., 2023). In terms of how mTOR is inhibited in CTD-induced liver autophagy and apoptosis, previous studies have found that DDIT4, a negative regulatory protein of mTOR, increases autophagy and apoptosis by inhibiting mTOR phosphorylation (Chen et al., 2016; Xu et al., 2021). DDIT4, as a stress protein, exists at low levels in most cells, including liver cells, and its expression is closely related to various cellular injuries (Chen et al., 2023). In non-alcoholic fatty liver disease, liver fibrosis, methionine-choline-deficient diet induced steatotic liver injury, bile duct ligation surgery induced cholestatic liver injury, and in CCl_4_ injection induced hepatotoxic injury, abnormal increase in DDIT4 expression (Dumas et al., 2020; Cho et al., 2021; Li et al., 2021). In our study, we also detected the expression levels of DDIT4 in the liver after CTD exposure. The results of qPCR, immunohistochemistry, and western blot showed that CTD exposure led to an abnormal increase in DDIT4 mRNA and protein expression. These results suggest that the CTD-induced autophagy and apoptosis of hepatocytes may be related to the upregulation of DDIT4 and inhibition of the mTOR pathway. Therefore, we speculate that the DDIT4/mTOR pathway plays an important role in CTD-induced liver injury.

To further explore the important role of the DDIT4/mTOR pathway in CTD-induced hepatocyte autophagy and apoptosis, we established a toxic model of L-O2 cells induced by CTD *in vitro*. The results of the CCK-8 assay showed that CTD induced toxicity in L-02 cells. According to the cell viability results after exposure to different concentrations of CTD for 6, 12, 24, and 36 h, and combined with the results of qPCR and western blotting for DDIT4, we selected CTD exposure concentrations of 7.5, 15, and 30 μmol/L and an exposure time of 24 h for follow-up experiments. Our results revealed that the DDIT4 protein expression in L-O2 cells also increased significantly after CTD exposure. The results of further detection of the mTOR pathway, autophagy, and apoptosis-related proteins in L-O2 cells were consistent with those in mice *in vivo*. Therefore, we speculate that the mechanism of CTD-induced autophagy and apoptosis in L-O2 cells may be related to the DDIT4/mTOR pathway.

To verify our hypothesis, we used RNAi to knock down the expression of DDIT4 and detected the expression of the mTOR pathway, autophagy, and apoptosis-related proteins. The results of western blot, flow cytometry, immunofluorescence, and transmission electron microscopy showed that knocking down DDIT4 expression significantly increased the expression of mTOR and p-mTOR and significantly inhibited CTD-induced autophagy and apoptosis of L-O2 cells. Furthermore, CTD-induced liver cell autophagy and apoptosis was increased after activating the mTOR pathway with the autophagy agonist Rap. The use of the autophagy inhibitor 3-MA to inhibit the mTOR pathway can alleviate CTD-induced liver cell autophagy and apoptosis. Taken together, these results suggest that CTD-induced autophagy and apoptosis in L-O2 cells are achieved through the DDIT4/mTOR pathway.

This study has some limitations that warrant discussion: 1) our *in vivo* experiments only positively prove that CTD-induced liver injury is related to autophagy and apoptosis of the DDIT4/mTOR pathway, while there remains a lack of negative evidence, such as verification at the gene knockout animal level; 2) there is insufficient evidence to prove that autophagy and apoptosis are the main mechanisms of CTD-induced hepatocyte injury; and 3) it remains unclear how CTD regulates the upregulation of DDIT4 expression and leads to autophagy and apoptosis of hepatocytes. We plan to conduct further research on these aspects in the future.

## 5 Conclusion

**FIGURE 10:**
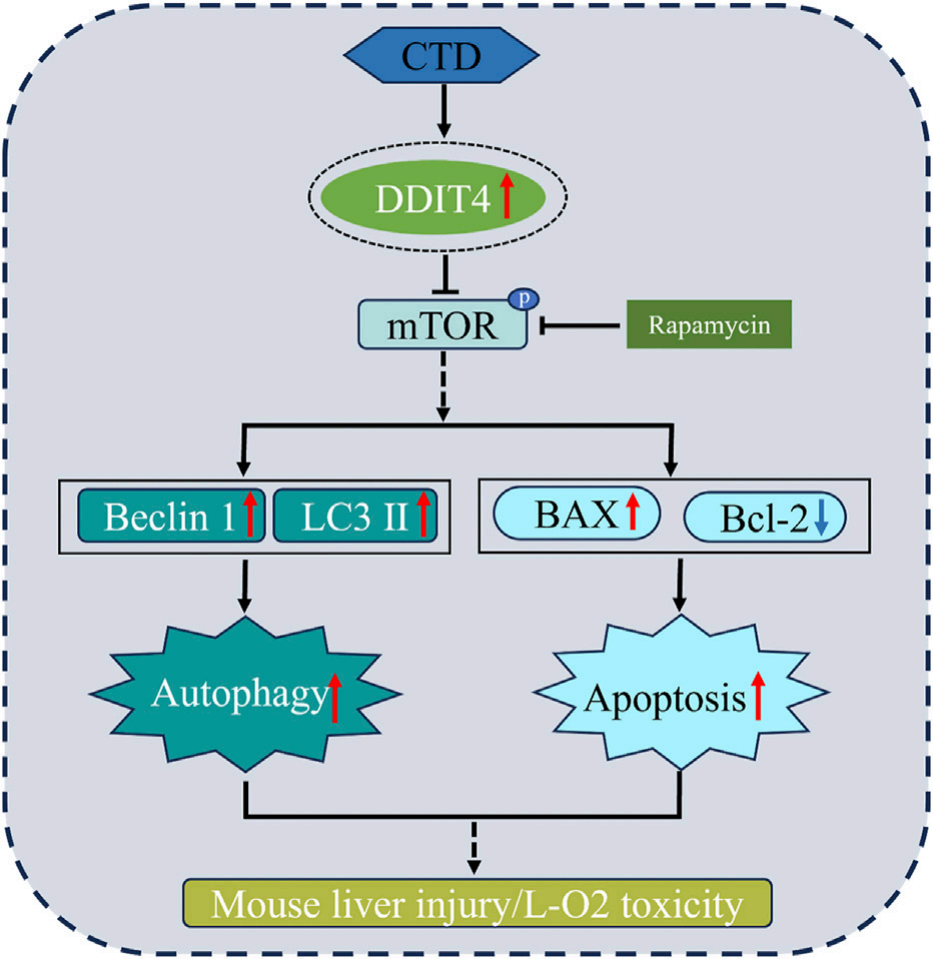
Toxic mechanism of CTD induced liver cell damage.

In summary, through *in vivo* and *in vitro* cell experiments, we proved that the molecular mechanism of liver injury and L-O2 cytotoxicity in mice induced by CTD may be mediated through increasing DDIT4 expression levels, thereby inhibiting mTOR phosphorylation and increasing autophagy and apoptosis. Autophagy and apoptosis of hepatocytes induced by CTD could be alleviated to a certain extent by knocking down DDIT4 or inhibiting the mTOR pathway (Figure 10).

## Data Availability

The datasets presented in this study can be found in online repositories. The names of the repository/repositories and accession number(s) can be found in the article/Supplementary Material.
