# Supplementary material for: DDIT4/mTOR signaling pathway mediates cantharidin-induced hepatotoxicity and cellular damage
Source: Front Pharmacol. 2024 Nov 5;15:1480512. doi: 10.3389/fphar.2024.1480512 (PMC11573530; doi:10.3389/fphar.2024.1480512)
Supplement: Supplementary file 1 [file Table1.pdf]

Gene	Primer	Sequence (5'-3')	PCR Products
GAPDH	Forward	TCAAGAAGGTGGTGAAGCAGG	115bp
	Reverse	TCAAAGGTGGAGGAGTGGGT	
DDIT4	Forward	CCATTCAAGCGGCAGGAC	172bp
	Reverse	GACGAGGGCGAAGAGGA	
