# Supplementary figures and images for: DDIT4/mTOR signaling pathway mediates cantharidin-induced hepatotoxicity and cellular damage
Source: Front Pharmacol. 2024 Nov 5;15:1480512. doi: 10.3389/fphar.2024.1480512 (PMC11573530; doi:10.3389/fphar.2024.1480512)

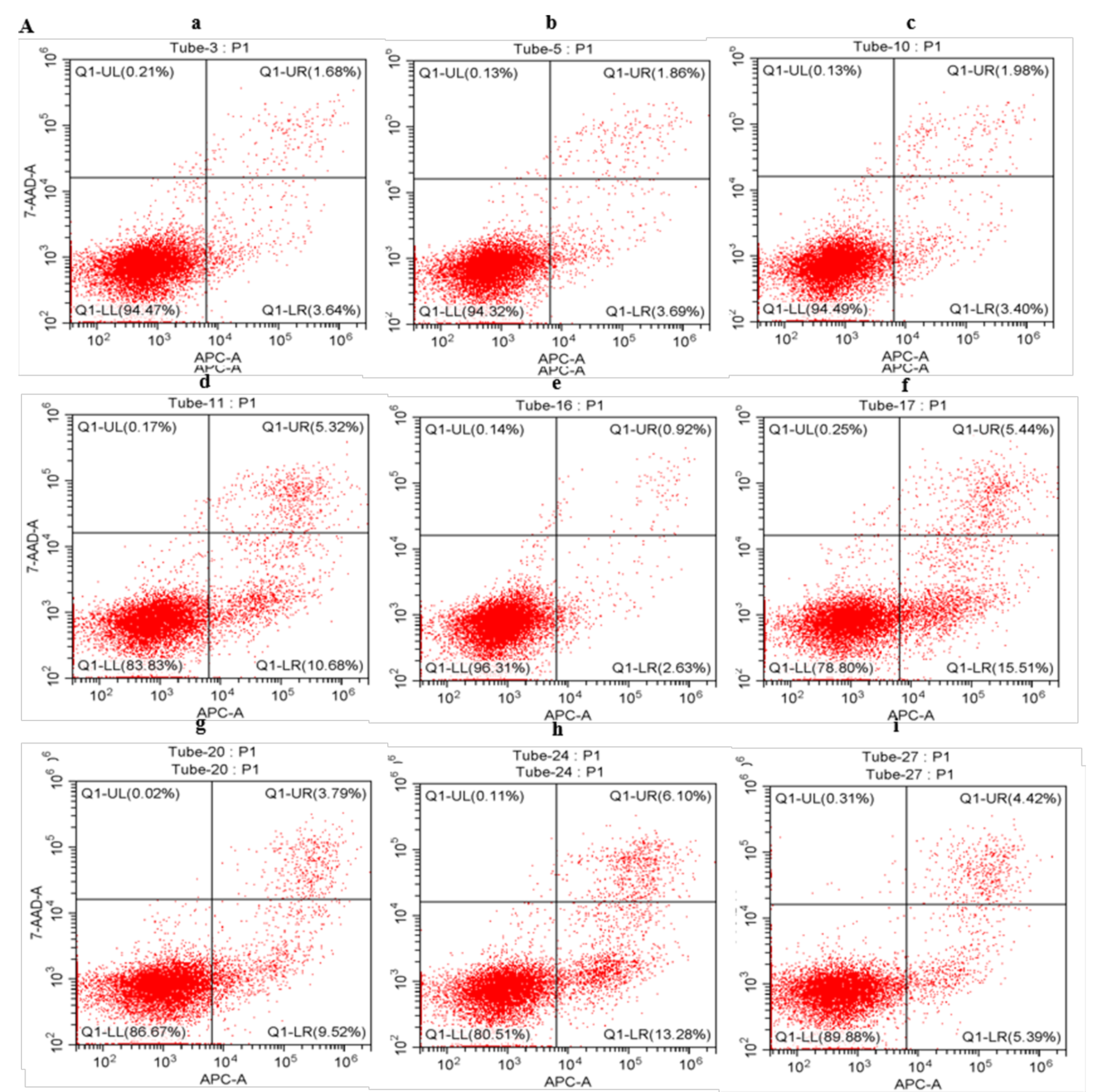

Supplement: Supplementary file 2 [file Image2.tif]

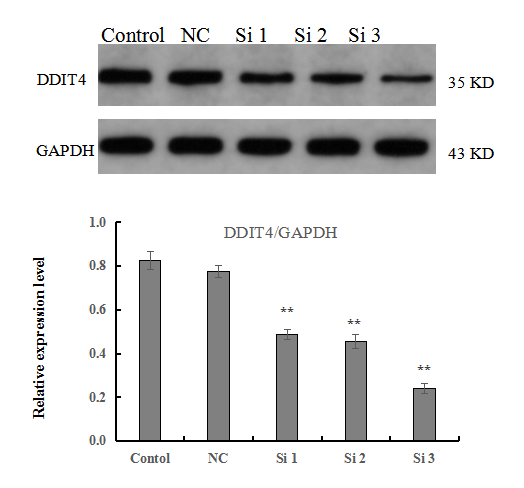

Supplement: Supplementary file 3 [file Image1.png]
